# Cytogenetic and developmental toxicity of bisphenol A and bisphenol S in *Arbacia lixula* sea urchin embryos

**DOI:** 10.1007/s10646-022-02568-w

**Published:** 2022-07-15

**Authors:** Raja Rezg, Rahime Oral, Serkan Tez, Bessem Mornagui, Giovanni Pagano, Marco Trifuoggi

**Affiliations:** 1 University of Monastir, ISBM, Biolival LR-14ES06, TN-5000 Monastir, Tunisia; 2 Faculty of Fisheries, Ege University, TR-35100 İzmir, Turkey; 3 Faculty of Sciences of Gabes, LR-18ES36, University of Gabes, TN-6072 Zrig, Gabes Tunisia; 4 Department of Chemical Sciences, Federico II Naples University, I-80126 Naples, Italy

**Keywords:** Bisphenol A, Bisphenol S, Developmental defects, Cytogenetic toxicity

## Abstract

Bisphenol S (BP-S) is one of the most important substitutes of bisphenol A (BP-A), and its environmental occurrence is predicted to intensify in the future. Both BP-A and BP-S were tested for adverse effects on early life stages of *Arbacia lixula* sea urchins at 0.1 up to 100 µM test concentrations, by evaluating cytogenetic and developmental toxicity endpoints. Embryonic malformations and/or mortality were scored to determine embryotoxicity (72 h post-fertilization). It has been reported in academic dataset that bisphenols concentration reached μg/L in aquatic environment of heavily polluted areas. We have chosen concentrations ranging from 0.1–100 μM in order to highlight, in particular, BP-S effects. Attention should be paid to this range of concentrations in the context of the evaluation of the toxicity and the ecological risk of BP-S as emerging pollutant. Cytogenetic toxicity was measured, using mitotic activity and chromosome aberrations score in embryos (6 h post-fertilization). Both BP-A and BP-S exposures induced embryotoxic effects from 2.5 to 100 µM test concentrations as compared to controls. Malformed embryo percentages following BP-A exposure were significantly higher than in BP-S-exposed embryos from 0.25 to 100 µM (with a ~5-fold difference). BP-A, not BP-S exhibited cytogenetic toxicity at 25 and 100 µM. Our results indicate an embryotoxic potential of bisphenols during critical periods of development with a potent rank order to BP-A vs. BP-S. Thus, we show that BP-A alternative induce similar toxic effects to BP-A with lower severity.

## Introduction

Bisphenol-A (BP-A) is an industrial chemical that has been used extensively to produce certain plastics and resins (Corrales et al. 2015). Current literature has raised concern about BP-A’s implications in several human chronic diseases (Rezg et al. 2014) and/or ecotoxicological complications (Corrales et al. 2015). These toxicologic impacts prompted different authorities to interdict this plasticizer from different industrial applications. Several countries have substituted the parental analog with bisphenol S (BP-S) under the “BP-A-free” label to indicate the safety of new products and reassure the consumer. However, the recent literature raised some doubts about the safety of “BPA-free” plastic products and has raised concern about their possible physiological disruptor properties and/or ecotoxicological effects (Mornagui et al. 2019; 2022; Qiu et al. 2019; Rezg et al. 2018; 2019; Wu et al. 2018; Wan et al. 2018; Zhou et al. 2019). BP-S is used in consumer products present in daily life such as food containers, canned foods, personal care products, paper products, manufactured plastics, and in many other industrial applications (Liao et al. 2012; Liao and Kannan 2014). Although the impact of microplastics and BP-A on marine wildlife is reported (Shahul Hamid et al. 2018; Xu et al. 2020), the adverse effects of BP-A alternatives as emergent pollutants are less well understood.

Bisphenols pass in aquatic environments through effluents discharged from wastewater treatment (when they are not completely removed before discharge), as well as directly from manufacturing industries, leachate discharges, and degradation of plastic litter (Corrales et al. 2015; Ying et al. 2009). Recently, BP-A and BP-S were detected as the predominant molecules in effluents of wastewater treatment plants in the US (Xue and Kannan 2019). Furthermore, BP-S has been detected in aquatic organisms and surface water samples from major rivers in many countries reaching, e.g., 7.2 μg/L in Adar, India (Yamazaki et al. 2015). As the usage of BP-A is predicted to decline further, environmental emissions of BP-S are likely to intensify in the future (Liu et al. 2021; Yu et al. 2015).

Sea urchins are an ecologically relevant animal group, and a valuable model frequently used for toxicity bioassays (Goldstone et al. 2006; Oral et al. 2017; Pagano et al. 2017). To the best of our knowledge, no data in the literature describes the toxicity of BP-S on sea urchins embryos. Thus, the aim of this study was to evaluate embryotoxicity and cytogenetic toxicity for both BP-A and BP-S in sea urchin embryos.

## Materials and methods

### Chemicals

Bisphenol A (BP-A; 4,4′-Isopropylidenediphenol; CAS 80-05-7, Purity 99%) and Bisphenol S (BP-S; 4,4′-Sulfonyldiphenol; CAS 80-09-1, Purity 98%) were obtained from Sigma-Aldrich Co.

### Sea urchins

*A. lixula*, which is distributed in shallow rocky reefs all along the Mediterranean coasts and are important grazers in sublittoral benthic communities, was used as test organism (Guidetti and Mori 2005). Specimens were collected by hand from the coastal side in Seferihisar, Izmir, Turkey (38.152331, 26.823245). Twenty liters of seawater were bottled from the sea urchin habitat. Specimens and water samples were transferred to the laboratory in icebox, then water samples were filtered with a 0.45 µm filter. Cytogenetic and developmental toxicity assays were carried out as described previously (Oral et al. 2017; Pagano et al. 2017). Cytogenetic toxicity tests were completed in polystyrene test beakers and contained 3 replicates whereas embryotoxicity tests were carried out in 6 replicates.

The choice of test concentrations was made according to Bošnjak et al. (2014) and based on the prediction that environmental emissions of BP-S are likely to intensify in the future (Liu et al. 2021; Yu et al. 2015). For this purpose, we selected concentrations ranging from 0.1 to 100 μM. Thus, the test concentrations of both chemicals were 0.1, 0.25, 1, 2.5, 10, 25, and 100 µM for both developmental and cytogenetic toxicity experiments.

Developmental and cytogenetic toxicity control groups consisted of untreated and healthy embryos (30 embryos/ml) in 10 ml of filtered seawater. Test chemicals were dissolved in dimethyl sulfoxide (DMSO), therefore a DMSO (0.1% v:v) control group for each test was applied as well.

### Embryological analysis

**Fig. 1:**
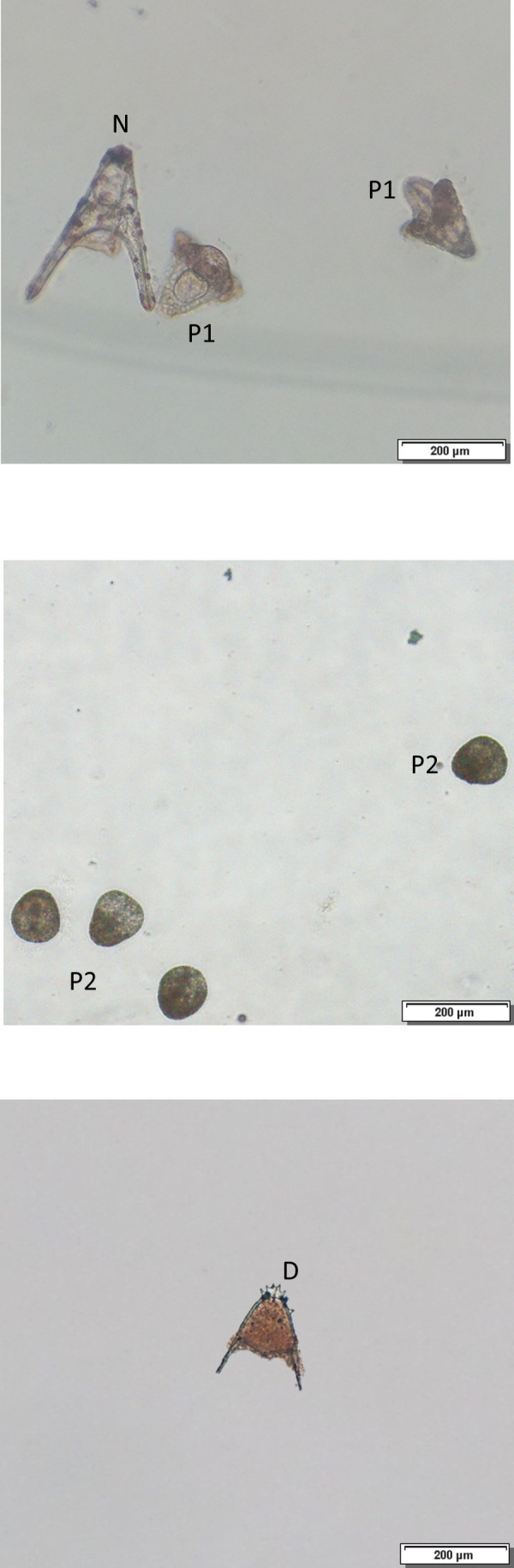
Embryonic malformations N: Normal pluteus, P1: pluteus with skeletal malformations, P2: blockage at pre-pluteus stages. D: early embryonic death

For embryotoxicity tests, BP-A or BP-S were placed at the bottom of each culture plate well [Falcon™ Tissue Culture Plates (6 wells, 10 ml/well)], and then suspended in 9 ml FSW. Thereafter, 1 ml of zygotes (10 min post-fertilization, p-f) was added to BP-A or BP-S and incubated at 18 °C in the dark for 72 h. After a 72-h incubation, 10^−4^ M chromium sulfate was added to the culture wells and the larvae were scored on an inverted microscope (100×) (Pagano et al. 2017). Embryonic/larval developmental defects were scored blind by trained readers in 100 random embryos of each test group to determine the embryotoxic effects of the test chemicals, as classified in Fig. 1: **N:** Normally developed plutei; **P1:** Malformed pluteus (skeletal and/or gastrointestinal malformations); **P2:** Developmental arrest at abnormal blastula/gastrula stage (pre-pluteus stage blockage). Developmental defects were calculated (%DD) = (P1 + P2). Another scored endpoint consists of the observation of dead plutei and dead pre-larval (or pre- hatching) embryos (**D:** early embryonic death). Thus, developmental defects and mortality were determined referring to the sum P1 + P2 + D.

### Cytogenetic analysis

**Fig. 2:**
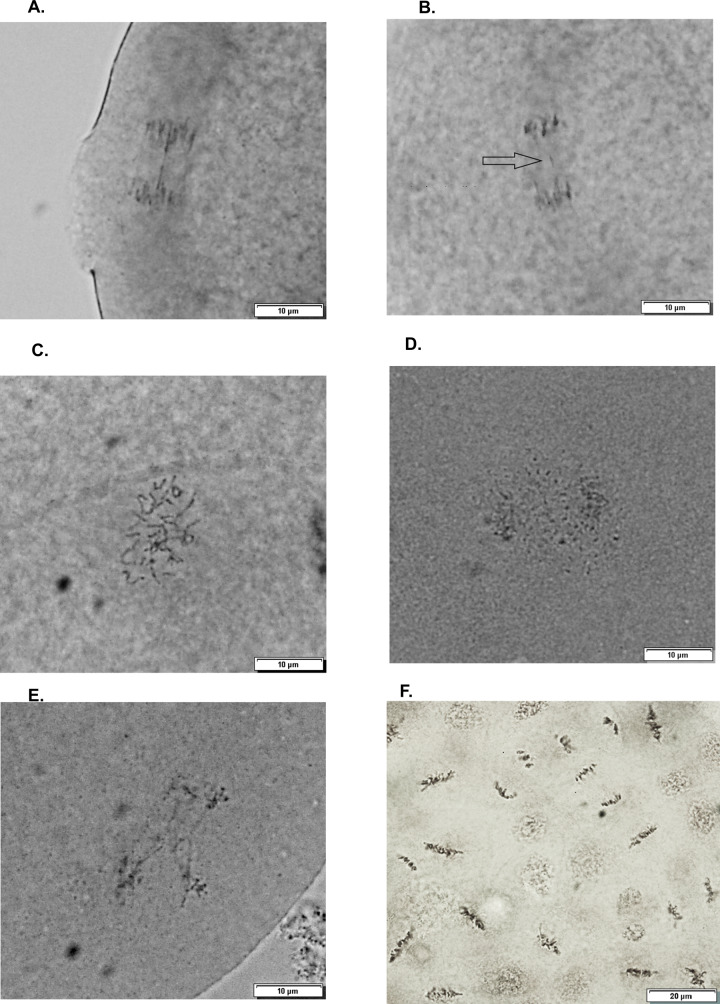
Mitotic aberrations **A** chromosome bridge, **B** lagging chromosome, **C** scattered, **D** fractured, **E** multipolar spindle. **F** normal mitosis

Cytogenetic tests were carried out 6 h p-f and the embryos were fixed in Carnoy’s solution (ethanol, chloroform, acetic acid; 6:3:1 V:V:V). Fixative was replaced with absolute ethanol right after fixation. 24 h after fixation, absolute ethanol was renewed and the samples were ready to be observed under a light microscope (1000×) with oil immersion. Mitotic activity (numbers of metaphase and anaphase) and chromosome aberrations (chromosome bridges, lagging chromosomes, multipolar spindles, free chromosome sets, fragmented chromosomes) as shown in Fig. 2, were scored in each embryo, thus allowing to assess both quantitative endpoints and mitotic anomalies.

### Statistical analysis

All datasets gathered from the bioassays were statistically analyzed in IBM SPSS v20. Results of bioassays are given as mean ± standard error in the charts. Homogeneity of variances was checked by Levene’s test. Differences between each concentration group and the controls were determined by two-tailed Independent Samples *t*-test. A normality test was performed and the significance of the difference among the groups was evaluated by One-way Analysis of Variance (ANOVA) with Tukey’s HSD and Tamhane’s T2 post-hoc tests. Kruskal-Wallis and Mann-Whitney U Tests were applied where ANOVA assumptions were not fulfilled. Differences were considered significant when *p* < 0.05.

## Results

### Embryotoxicity

**Fig. 3:**
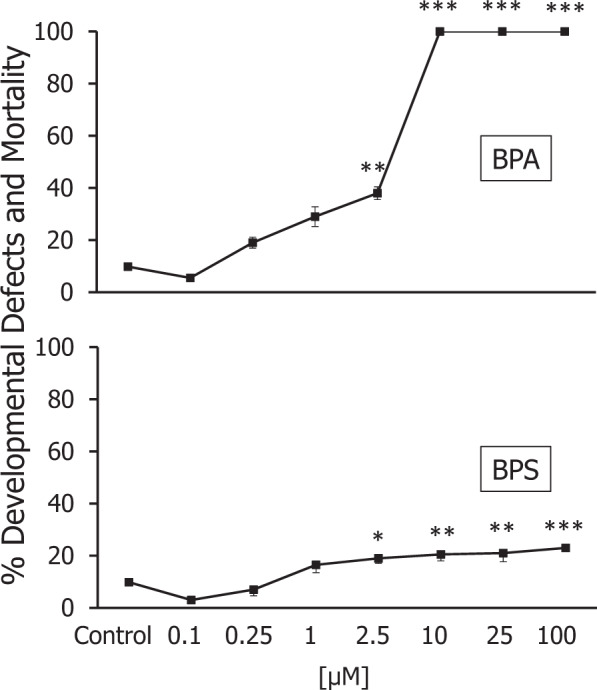
Average affected embryo percentages in embryotoxicity tests after BP-A or BP-S treatment (*p < 0.05, **p < 0.01 vs control, Tamhane’s, Tukey’s)

BP-A started to induce embryotoxic effects with 29% of malformed embryos at 1 µM concentration, as shown in Fig. 3. Compared to the control groups, malformed embryo percentages significantly differed at 2.5 µM (*p* < 0.01, Tamhane’s). 10, 25, and 100 µM concentrations of BP-A affected all embryos in the test groups (*p* < 0.001, Tamhane’s). Malformed embryo rates in embryos exposed to BP-S showed significant differences at 2.5 µM compared to the control groups (p < 0.05, Tukey’s). 10 and 25 µM concentrations were at a close embryotoxic level (20.5 to 21%) and differed from the controls (*p* < 0.01, Tukey’s). Malformed embryo rates raised to 23% at 100 µM concentration (*p* < 0001, Tukey’s). Malformed embryo percentages in BP-A vs. BP-S significantly differed at 0.25 µM (*p* < 0.01), 1 µM (*p* < 0.05), 2.5 µM (*p* < 0.01), 10 µM (*p* < 0.001), 25 µM (*p* < 0.001) and 100 µM (*p* < 0.001) (Student’s t tests). EC_50_ was calculated based on the nominal concentrations and it was found as 3.48 µM (95% Confidence Interval: 1.84 to 6.53 µM) for BP-A and not calculated for BP-S (because with tested concentrations, data do not reach a maximal effect). Altogether, developmental toxicity of BP-S was significantly lower than BP-A-induced developmental toxicity.

### Cytogenetic toxicity

**Fig. 4:**
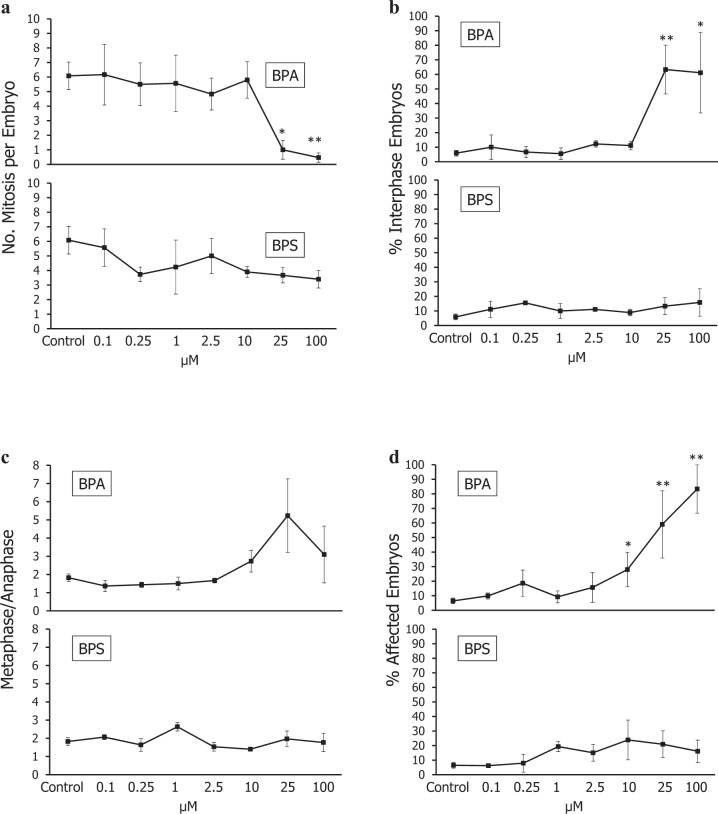
Cytogenetic toxicity after BP-A or BP-S exposure in *A. lixula* sea urchin embryos. **a** Mean of no.mitoses per embryo (*p < 0.05; **p < 0.01; ***p < 0.001 vs control, Tukey’s). **b** Percentages of interphase embryos (*p < 0.05; **p < 0.01; ***p < 0.001 vs control, Student’s *t* and Mann-Whitney U tests). **c** Metaphase/Anaphase ratio (*p < 0.05; **p < 0.01; ***p < 0.001 vs control, Student’s *t*). **d** Percentage of affected embryos (percent embryos having ≥1 mitotic aberrations) (*p < 0.05; **p < 0.01; ***p < 0.001 vs control, Tukey’s)

The cytogenetic results for BP-A plasticizer and its substitute BP-S are shown in Fig. 4. Mitotic activity in the embryos exposed to BP-A was inhibited at 25 (*p* < 0.05, Student’s t) and 50 µM (*p* < 0.01, Student’s t) concentrations. At the concentrations of 25 and 50 µM, mitotic activity significantly differed for BP-A and BP-S (*p* < 0.05, Student’s t) (Fig. 4a). Also the data in Fig. 4b showed that the number of embryos lacking mitotic figures (% Interphase Embryos, IE) differed at 25 to 50 µM BP-A vs. Control, and significantly above the corresponding IE values induced by BP-S (*p* < 0.05, Student’s t). As shown in Fig. 4d, a significant difference was observed in average total mitotic aberrations in embryos exposed to 25 to 50 µM BP-A compared to controls (*p* < 0.05, Mann-Whitney U test), and compared to embryos exposed to BP-S (*p* < 0.05, Mann-Whitney U test).

## Discussion

Several studies have reported on pleiotropic toxic effects of BP-A in aquatic vertebrates and invertebrates at environmental doses (Canesi and Fabbri 2015; Crain et al. 2007; Kang et al. 2007). BP-A-induced embryotoxicity was noted previously, in sea urchins (Cakal Arslan and Parlak 2008), in zebrafish (Tse et al. 2013), in Xenopus (Gibert et al. 2011), and rodents (Chen et al. 2013).

It has been reported that BP-A can alter echinoderm physiology, reproduction, and development at environmental concentrations (Bošnjak et al. 2014; Roepke et al. 2005), which can reach 17.2 μg/L (Crain et al. 2007). BP-A can induce aberrant karyokinesis (division of the cell nucleus), leading to defective embryo development through the first cell division and retardation, along with general errors in cytoskeletal functioning in mitosis (Bošnjak et al. 2014).

The present report confirms BP-A-induced developmental and cytogenetic toxicity, while the replacement chemical (BP-S) failed to alter *A. lixula* early life stages. BP-A is more potent than BP-S in particular, at 10, 25, and 100 μM (~5 fold), indicating the sensitivity of *A. lixula* embryos to these specific bisphenols during a critical developmental period. Analogous effects were also noted within *Daphnia magna* and in Zebrafish embryos and larvae (Liu et al. 2021). Thus, we suggest that BP-S raises fewer, if any, environmental problems with its growing use in replacing BP-A.

The toxicity order for different bisphenols reflects that they may operate *via* distinct mechanisms.

It has been reported in academic dataset that bisphenols concentration reached μg/L in aquatic environment of heavily polluted areas (Liu et al. 2021). For example, levels of BP-S detected in surface waters of the Adyar River and Buckingham Canal in India have been found to reach to range from non-detectable to 7.20 μg/L and 0.058 to 2.1 μg/L respectively. For BP-A it can reach 17.2 μg/L (Crain et al. 2007). It has been also cited that bisphenol environmentally relevant concentrations are from 0.1 to 1000 μg/L (Qiu et al. 2018). Before 2013, BP-S had been detected in freshwater and sewage sludge, but rarely found in marine surface sediment. However, recent literature showed that BP-S concentration in aquatic environments started to increase progressively (Wu et al. 2018). This observation may indicate that BP-S compounds begin to be extensively used all over the world at different degrees with countries (Liu et al. 2021).

In addition, attention should be paid to the range of concentrations from 0.1–100 µM to develop environmental predictions and risk management because it has been reported that the usage of BP-A is predicted to decline further, and environmental emissions of BP-S are likely to increase in the future (Liu et al. 2021; Yu et al. 2015). Besides, BP-S is less biodegradable than BP-A in aquatic environments, which may lead to its accumulation in the biota (Danzl et al. 2009; Herrero et al. 2018). Thus, in this experimental protocol, we have chosen concentrations ranging from 0.1–100 μM in order to highlight, in particular, BP-S effects. It could be important in the context of evaluation of toxicity and ecological risk of BP-S as emerging pollutant.

Data indicate that BP-S did not exert cytogenetic toxicity at all test concentrations as compared to controls, whereas BP-A can induce cytogenetic anomalies in particular at high concentrations, 25 and 50 μM. In accordance with our data, several studies have reported that BP-A can induce DNA damage as well as structural and numerical chromosomal aberrations in vitro (Santovito et al. 2018; Xin et al. 2015) and in vivo (Izzotti et al. 2009). A recent study describes no cytogenetic effects for both BP-A and BP-S in human HepG2 cells (Hercog et al. 2020). Also, it has been reported that BP-S, compared to BPA, has a lower acute toxicity, similar or less endocrine disruption, similar neurotoxicity, and immunotoxicity, and lower reproductive and developmental toxicity (Qiu et al. 2018). On the other hand, to date there is a lack of information on the effects of BP-S on invertebrates cytogenetic, just Herrero et al. (2018) reported negative effects of BP-S on the transcriptional rate of genes in the model species *Chironomus riparius* on the whole.

### Potential mechanisms for toxicity during larval development

It has been found a relationship between species relatedness and the estrogen agonist mode of action in BP-A-induced developmental alterations. Thus, a cross-species mode of a action via estrogen signaling have been shown leading to physiological changes in vertebrates (fish and mammals) and invertebrates (U.S. EPA 2005). Although research on endocrine disruptors and echinoderm has not been abundant, the existence of species-specific sensitivity in urchin species against BP-A and several other endocrine-disrupting compounds, on larval stage development was reported (Roepke et al. 2005). The authors concluded that EDCs could act with different mode of action (other than estrogen signaling), leading to differential response and sensitivity in embryos of each species of sea urchin. Thus, yet the molecular mechanisms or modes of action underlying bisphenols-induced developmental and cytogenetic toxicity is poorly understood in invertebrates due to the pleiotropic effects. It is instructive to offer some plausible mechanistic hypotheses:

- Endocrine disruption: While current knowledge of echinoderm endocrinology is still limited and not well understood, early evidence has reported that echinoderms physiology acts via vertebrate-like hormones (such as steroids) (Sugni et al. 2007) and it has been reported that thyroid hormones are implicated in Echinoderm metamorphosis process (Heyland et al. 2005). Also, a genomic analysis of sea urchin nervous system has been elucidated at least 37 putative G-protein-coupled peptide receptors and peptide hormones (Burke et al. 2006). Thus, in sea urchin embryos, hormones may be acts on specific targets larval development and any disruption could induce negative impact.
- Changes in the expression of a whole host of genes/gene networks, which may impact successful early developmental organisation and growth of larvae (Bošnjak et al. 2014).
- Lipid peroxidation and oxidative stress to DNA resulting in developmental impacts and toxic effects of both BP-A and BP-S as proved with transcriptome approach in zebrafish model (Yang et al. 2021).
- Epigenetic changes such as alterations in DNA methylation (Qin et al. 2021).

## Conclusions

This study evaluated the effects of BPA and BPS on sea urchin embryos providing some data support for their potential ecological risks. Taken together, our results indicate an embryotoxic potential of both BP-A and its substitute BP-S during critical periods of sea urchin development with a potent rank order to BP-A vs. BP-S. We thus show that BP-A alternative, BP-S induces lower toxic effects than BP-A with significantly lower severity, though suggesting possibly concerns regarding the use of this BP-A alternative. Ultimately, several studies have shed light on embryotoxic potential of BP-A in humans, vertebrates, and invertebrates and reveal concern about the Safety of BP-A substitutes. Since the use of BPA alternative compounds is increasing, further monitoring data of the water environment and chronic toxicity in various aquatic organisms appears to be necessary.

## References

[CR1] Bošnjak I, Borra M, Iamunno F, Benvenuto G, Ujević I, Bušelić I, Roje-Busatto R, Mladineo I (2014). Effect of bisphenol A on P-glycoprotein-mediated efflux and ultrastructure of the sea urchin embryo. Aquat Toxicol.

[CR2] Burke RD, Angerer LM, Elphick MR, Humphrey GW, Yaguchi S, Kiyama T, Thorndyke MC (2006). A genomic view of the sea urchin nervous system. Dev Biol.

[CR3] Cakal Arslan O, Parlak H (2008). Effects of bisphenol A on the embryonic development of sea urchin *(Paracentrotus lividus)*. Environ Toxicol.

[CR4] Canesi L, Fabbri E (2015). Environmental effects of BPA: Focus on aquatic species. Dose-Response.

[CR5] Chen X, Xu B, Han X, Mao Z, Talbot P, Chen M, Du G, Chen A, Liu J, Wang X, Xia Y (2013). Effect of bisphenol A on pluripotency of mouse embryonic stem cells and differentiation capacity in mouse embryoid bodies. Toxicol in Vitro.

[CR6] Corrales J, Kristofco LA, Steele WB, Yates BS, Breed CS, Williams ES, Brooks BW (2015). Global assessment of bisphenol A in the environment: Review and analysis of its occurrence and bioaccumulation. Dose Response.

[CR7] Crain DA, Eriksen M, Iguchi T, Jobling S, Laufer H, LeBlanc GA, Guillette LJ (2007). An ecological assessment of bisphenol-A: Evidence from comparative biology. Reprod Toxicol.

[CR8] Danzl E, Sei K, Soda S, Ike M, Fujita M (2009). Biodegradation of bisphenol A, bisphenol F and bisphenol S in seawater. Int J Environ Res Publ Health.

[CR9] Gibert Y, Sassi-Messai S, Fini JB, Bernard L, Zalko D, Cravedi JP, Balaguer P, Andersson-Lendahl M, Demeneix B, Laudet V (2011). Bisphenol A induces otolith malformations during vertebrate embryogenesis. BMC Devel Biol.

[CR10] Goldstone JV, Hamdoun A, Cole BJ, Howard-Ashby M, Nebert DW, Scally M, Dean M, Epel D, Hahn ME, Stegeman JJ (2006). The chemical defensome: environmental sensing and response genes in the *Strongylocentrotus purpuratus* genome. Devel Biol.

[CR11] Guidetti P, Mori M (2005). Morpho-functional defences of Mediterranean sea urchins, *Paracentrotus lividus* and *Arbacia lixula*, against fish predators. Mar Biol.

[CR12] Hercog K, Štern A, Maisanaba S, Filipič M, Žegura B (2020). Plastics in cyanobacterial blooms-genotoxic effects of binary mixtures of cylindrospermopsin and bisphenols in HepG2 cells. Toxins.

[CR13] Herrero Ó, Aquilino M, Sánchez-Argüello P, Planelló R (2018). The BPA-substitute bisphenol S alters the transcription of genes related to endocrine, stress response and biotransformation pathways in the aquatic midge *Chironomus riparius* (Diptera, Chironomidae). PLoS One.

[CR14] Heyland A, Hodin J, Reitzel AM (2005). Hormone signaling in evolution and development: a non‐model system approaches. Bioessays.

[CR15] Izzotti A, Kanitz S, D’Agostini F, Camoirano A, De Flora S (2009). Formation of adducts by bisphenol A, an endocrine disruptor, in DNA in vitro and in liver and mammary tissue of mice. Mutat Res.

[CR16] Kang JH, Asai D, Katayama Y (2007). Bisphenol A in the aquatic environment and its endocrine-disruptive effects on aquatic organisms. Crit Rev Toxicol.

[CR17] Liao C, Liu F, Kannan K (2012). Bisphenol S, a new bisphenol analogue, in paper products and currency bills and its association with bisphenol A residues. Environ Sci Technol.

[CR18] Liao C, Kannan K (2014). A survey of alkylphenols, bisphenols, and triclosan in personal care products from China and the United States. Arch Environ Contam Toxicol.

[CR19] Liu J, Zhang L, Lu G, Jiang R, Yan Z, Li Y (2021). Occurrence, toxicity and ecological risk of Bisphenol A analogues in aquatic environment - A review. Ecotoxicol Environ Saf.

[CR20] Mornagui B, Rezg R, Repond C, Pellerin L (2019). Effects of bisphenol S, a major substitute of bisphenol A, on neurobehavioral responses and cerebral monocarboxylate transporters expression in mice. Food Chem Toxicol.

[CR21] Mornagui B, Rezg R, Repond C, Pellerin L, (2022) Bisphenol S favors hepatic steatosis development via an upregulation of liver MCT1 expression and an impairment of the mitochondrial respiratory system. J Cell Physiol 10.1002/jcp.30771 10.1002/jcp.30771 35561261

[CR22] Oral R, Pagano G, Siciliano A, Gravina M, Palumbo A, Castellano I, Oriana Migliaccio O, Thomas PJ, Guida M, Tommasi F, Trifuoggi M (2017). Heavy rare earth elements affect early life stages in *Paracentrotus lividus* and *Arbacia lixula* sea urchins. Environ Res.

[CR23] Pagano G, Guida M, Trifuoggi M, Thomas PJ, Palumbo A, Romano G, Oral R (2017). Sea urchin bioassays in toxicity testing: I. Inorganics, organics, complex mixtures and natural products. Expert Opin Environ Biol.

[CR24] Qiu W, Yang M, Liu S, Lei P, Hu L, Chen B, Wu M, Wang KJ (2018). Toxic effects of Bisphenol S showing immunomodulation in fish macrophages. Environ Sci Technol.

[CR25] Qiu W, Zhan H, Hu J, Zhang T, Xu H, Wong M, Xu B, Zheng C (2019). The occurrence, potential toxicity, and toxicity mechanism of bisphenol S, a substitute of bisphenol A: A critical review of recent progress. Ecotoxicol Environ Saf.

[CR26] Qin T, Zhang X, Guo T, Yang T, Gao Y, Hao W, Xiao X (2021). Epigenetic alteration shaped by the environmental chemical bisphenol A. Front genetics.

[CR27] Rezg R, El-Fazaa S, Gharbi N, Mornagui B (2014). Bisphenol A and human chronic diseases: current evidences, possible mechanisms, and future perspectives. Environ Int.

[CR28] Rezg R, Abot A, Mornagui B, Aydi S, Knauf C (2018). Effects of Bisphenol S on hypothalamic neuropeptides regulating feeding behavior and apelin/APJ system in mice. Ecotoxicol Environ Saf.

[CR29] Rezg R, Abot A, Mornagui B, Knauf C (2019). Bisphenol S exposure affects gene expression related to intestinal glucose absorption and glucose metabolism in mice. Environ Sci Pollut Res Int.

[CR30] Roepke TA, Snyder MJ, Cherr GN (2005). Estradiol and endocrine disrupting compounds adversely affect development of sea urchin embryos at environmentally relevant concentrations. Aquat Toxicol.

[CR31] Santovito A, Cannarsa E, Schleicherova D, Cervella P (2018). Clastogenic effects of bisphenol A on human cultured lymphocytes. Hum Exp Toxicol.

[CR32] Shahul Hamid F, Bhatti MS, Anuar N, Anuar N, Mohan P, Periathamby A (2018). Worldwide distribution and abundance of microplastic: How dire is the situation?. Waste Manag Res.

[CR33] Sugni M, Mozzi D, Barbaglio A, Bonasoro F, Carnevali MD (2007). Endocrine disrupting compounds and echinoderms: new ecotoxicological sentinels for the marine ecosystem. Ecotoxicology.

[CR34] Tse W, Yeung B, Wan H, Wong C (2013). Early embryogenesis in zebrafish is affected by bisphenol A exposure. Biol Open.

[CR35] U.S. EPA (2005). A Cross-Species Mode Of Action (MOA) Information Assessment: A Case Study Of Bisphenol A (BPA).

[CR36] Wan Y, Xia W, Yang S, Pan X, He Z, Kannan K (2018). Spatial distribution of bisphenol S in surface water and human serum from Yangtze River watershed, China: Implications for exposure through drinking water. Chemosphere.

[CR37] Wu LH, Zhang XM, Wang F, Gao CJ, Chen D, Palumbo JR, Zeng EY (2018). Occurrence of bisphenol S in the environment and implications for human exposure: A short review. Sci Total Environ.

[CR38] Xin L, Lin Y, Wang A, Zhu W, Liang Y, Su X, Tian H (2015). Cytogenetic evaluation for the genotoxicity of bisphenol-A in Chinese hamster ovary cells. Environ Toxicol Pharmacol.

[CR39] Xu S, Ma J, Ji R, Pan K, Miao AJ (2020). Microplastics in aquatic environments: Occurrence, accumulation, and biological effects. Sci Total Environ.

[CR40] Xue J, Kannan K (2019). Mass flows and removal of eight bisphenol analogs, bisphenol A diglycidyl ether and its derivatives in two wastewater treatment plants in New York State, USA. Sci Total Environ.

[CR41] Yamazaki E, Yamashita N, Taniyasu S, Lam J, Lam PKS, Moon HB, Kannan K (2015). Bisphenol A and other bisphenol analogues including BPS and BPF in surface water samples from Japan, China, Korea and India. Ecotoxicol Environ Saf.

[CR42] Yang F, Zhao Z, Zhang H, Zhou L, Tao L, Wang Q (2021). Concentration-dependent transcriptome of zebrafish larvae for environmental bisphenol S assessment. Ecotox Environ Saf.

[CR43] Ying GG, Kookana RS, Kumar A, Mortimer M (2009). Occurrence and implications of estrogens and xenoestrogens in sewage effluents and receiving waters from South East Queensland. Sci Total Environ.

[CR44] Yu X, Xue J, Yao H, Wu Q, Venkatesan AK, Halden RU, Kannan K (2015). Occurrence and estrogenic potency of eight bisphenol analogs in sewage sludge from the U.S. EPA targeted national sewage sludge survey. J Hazard Mater.

[CR45] Zhou Z, Lei Y, We W, Zha Y, Jian Y, Wan N, Xiaofeng L, Chen X (2019). Association between prenatal exposure to bisphenol A and birth outcomes: A systematic review with meta-analysis. Medicine (Baltimore).

